# Retrospective analysis of arterial occlusive events in the PACE trial by an independent adjudication committee

**DOI:** 10.1186/s13045-021-01221-z

**Published:** 2022-01-06

**Authors:** James L. Januzzi, Joseph M. Garasic, Scott E. Kasner, Vickie McDonald, Mark C. Petrie, Jonathan Seltzer, Michael Mauro, Kevin Croce, Ellin Berman, Michael Deininger, Andreas Hochhaus, Javier Pinilla-Ibarz, Franck Nicolini, Dong-Wook Kim, Daniel J. DeAngelo, Hagop Kantarjian, Jing Xu, Tracey Hall, Shouryadeep Srivastava, Daniel Naranjo, Jorge Cortes

**Affiliations:** 1 Massachusetts General Hospital, 55 Fruit Street, Boston, MA USA; 2 University of Pennsylvania, Philadelphia, PA USA; 3 Barts Health NHS Trust, London, England; 4 University of Glasgow, Glasgow, Scotland; 5 ACI Clinical, Bala Cynwyd, PA USA; 6 Memorial Sloan Kettering Cancer Center, New York, NY USA; 7 Brigham and Women’s Hospital, Harvard Medical School, Boston, MA USA; 8 Huntsman Cancer Institute, The University of Utah, Salt Lake City, UT USA; 9 Universitätsklinikum Jena, Jena, Germany; 10 H. Lee Moffitt Cancer Center & Research Institute, Tampa, FL USA; 11 Centre Hospitalier Lyon-Sud, Pierre-Bénite, Lyon, France; 12 Catholic Hematology Hospital, Seoul St. Mary’s Hospital, Leukemia Research Institute, The Catholic University of Korea, Seoul, South Korea; 13 Dana-Farber Cancer Institute, Boston, MA USA; 14 The University of Texas MD Anderson Cancer Center, Houston, TX USA; 15 Millennium Pharmaceuticals, Inc., A Wholly Owned Subsidiary of Takeda Pharmaceutical Company Limited, Cambridge, MA USA; 16 Georgia Cancer Center, Augusta, GA USA

**Keywords:** Acute lymphoblastic leukemia, Chronic myeloid leukemia, Safety, Tyrosine kinase inhibitor

## Abstract

**Background:**

The phase 2 PACE (Ponatinib Ph+ ALL and CML Evaluation) trial of ponatinib showed robust long-term benefit in relapsed Philadelphia chromosome-positive (Ph+) leukemia; arterial occlusive events (AOEs) occurred in ≥ 25% of patients based on investigator reporting. However, AOE rates vary depending on the definitions and reporting approach used.

**Methods:**

To better understand clinically relevant AOEs with ponatinib, an independent cardiovascular adjudication committee reviewed 5-year AOE data from the PACE trial according to a charter-defined process and standardized event definitions.

**Results:**

A total of 449 patients with chronic myeloid leukemia (CML) or Ph+ acute lymphoblastic leukemia (ALL) received ponatinib (median age 59 y; 47% female; 93% ≥ 2 prior tyrosine kinase inhibitors (TKIs); median follow-up, 37.3 months). The adjudicated AOE rate (17%) was lower than the non-adjudicated rate (i.e., rate before adjudication; 25%). The only adjudicated AOE in > 2% of patients was peripheral arterial occlusive disease (4%). Exposure-adjusted incidence of newly occurring adjudicated AOEs decreased over time. Patients with multiple baseline cardiovascular risk factors had higher adjudicated AOE rates than those without risk factors.

**Conclusions:**

This independent adjudication study identified lower AOE rates than previously reported, suggesting earlier overestimation that may inaccurately reflect AOE risk with ponatinib. This trial was registered under ClinicalTrials.gov identifier NCT01207440 on September 23, 2010 (https://clinicaltrials.gov/ct2/show/NCT01207440).

## Background

Ponatinib, a pan-BCR::ABL1 inhibitor, is an orally active third-generation tyrosine kinase inhibitor (TKI) designed to potently inhibit BCR::ABL1 with or without any point mutation, including *BCR::ABL1*^*T315I*^ [1]*.* In the pivotal phase 2 PACE (Ponatinib Ph+ ALL and CML Evaluation) trial, ponatinib demonstrated robust clinical activity with rapid, deep, and long-term responses, progression-free survival (PFS), and overall survival in patients with chronic-phase chronic myeloid leukemia (CP-CML), ≥ 90% of whom had failed treatment with ≥ 2 TKIs, regardless of the presence or absence of BCR::ABL1 mutations, including T315I [2, 3]. The 5-year results of the PACE trial confirmed the durability of these responses with a 5-year overall survival rate of 73% for CP-CML [3]. However, arterial occlusive events (AOEs) were reported by investigators in 25% in the overall population (serious AOEs, 20%) and 31% in the CP-CML population (serious AOEs, 26%) in the 5-year follow-up [3]. The exposure-adjusted incidence of newly occurring AOEs decreased from year 1 (15.8 patients with events per 100 patient-years in the total population) to year 5 (3.9 per 100 patient-years) [3]. The incidence of AOEs associated with ponatinib use has varied widely in subsequent reports. Two retrospective studies have reported an absence or very low incidence (6%) of AOEs [4, 5]. Other real-world studies have reported AOE rates ranging from 18 to 26% [6, 7]. Multiple factors may contribute to variability in reported AOE rates, including differences in patient populations, as well as differences in the clinical definitions used to identify and categorize vascular occlusive events. One of the most important factors is the lack of a standardized approach for defining and capturing AOEs with BCR::ABL1 TKIs.

The AOE incidence rate reported for PACE was based on a list of approximately 400 Medical Dictionary for Regulatory Activities (MedDRA) preferred terms developed by the sponsor. However, differences in the preferred terms used to define AOEs led to variability in AOE incidence rates. Some preferred terms included in the AOE analysis of PACE are highly sensitive for identification of potential AOEs but may not themselves indicate the occurrence of arterial occlusions, frequently including symptoms or descriptions rather than events; these include chest pain, cold hands, dysarthria, and poor peripheral circulation. This approach to characterize AOEs based on adverse event terms results in broadly capturing non-specific symptoms that may be associated with AOE rather than true AOEs and may thus overestimate the incidence of clinically meaningful events.

A clear understanding of clinically relevant AOE risk is imperative when characterizing the benefit-risk profile of ponatinib. Patients with CP-CML who become resistant to a second-generation BCR::ABL1 TKI, either with or without a *BCR::ABL1* gene mutation, generally experience low response rates and poor survival if treated with another second-generation TKI [8, 9]. Importantly, ponatinib is the only currently available TKI effective in patients with the *BCR::ABL1*^*T315I*^ mutation [3]. Therefore, the potential for improved survival and duration of response on ponatinib may outweigh the risk of AOEs [8, 9]. However, the lack of clear data regarding clinically meaningful AOEs has led to confusion about how to optimally use ponatinib to treat relapsed/refractory CML and Philadelphia chromosome-positive (Ph+) acute lymphoblastic leukemia (ALL) and, in some instances, avoidance in patients who could potentially benefit. To provide a more accurate characterization of AOE incidence with ponatinib, an independent adjudication committee of experts was convened to retrospectively adjudicate all AOE reports in the PACE trial in a standardized, rigorous manner.

## Methods

### PACE trial design

The phase 2 PACE trial (ClinicalTrials.gov identifier: NCT01207440) enrolled adults with CML or Ph+ ALL whose disease was resistant or intolerant to dasatinib or nilotinib, or who had the *BCR::ABL1*^*T315I*^ mutation regardless of prior TKI use [3]. All patients received ponatinib at a starting dose of 45 mg once daily (qd); dose reductions to 30 or 15 mg qd were applied per protocol (Table 1) to manage adverse events (AEs), or implemented proactively following recommendations from the sponsor in October 2013 in response to AOEs emerging as notable AEs. The trial has been completed; detailed methods are published [2, 3].

**Table 1:** Dose reduction recommendations (as of 2013)

*Dose reduction recommendations*
In October 2013, the following specific recommendations were formulated after discussions with the US FDA on evolving observations of arterial occlusive events in patients treated with ponatinib:
All chronic phase chronic myeloid leukemia (CP-CML) patients on study who already had achieved major cytogenetic response (MCyR) should have had their dose reduced to 15 mg daily, unless, in the judgment of the investigator, the benefit/risk analysis, taking into account the patient's disease characteristics, *BCR::ABL* mutation status, and the patient's cardiovascular risk justified treatment with a higher dose
All CP-CML patients on study who had not yet achieved MCyR should have had their dose reduced to 30 mg daily, unless, in the judgment of the investigator, the benefit/risk analysis, taking into account the patient's disease characteristics, *BCR::ABL* mutation status, and the patient's cardiovascular risk justified treatment with a higher dose
All acute phase chronic myeloid leukemia (AP-CML), blast phase chronic myeloid leukemia (BP-CML), and Ph+ acute lymphoblastic leukemia (ALL) patients on study should have had their dose reduced to 30 mg daily, unless, in the judgment of the investigator, the benefit/risk analysis, taking into account the patient's disease characteristics, *BCR::ABL* mutation status, and the patient's cardiovascular risk justified treatment with a higher dose
All patients who lost response at a lower dose may have their dose escalated (up to a maximum of 45 mg daily) as long as the dose was not lowered as a result of an adverse event (AE)

### Adjudication methods

All activities related to the adjudication of AOEs were conducted by ACI Clinical (Bala Cynwyd, PA), including the identification of an independent adjudication committee. ACI Clinical is a clinical research organization with expertise in Endpoint Adjudication and Data Monitoring Committees to support safety decisions around clinical development programs. ACI Clinical was contracted by the sponsor; adjudication activities were not part of the PACE trial.

#### Identification of AEs for adjudication

**Fig. 1:**
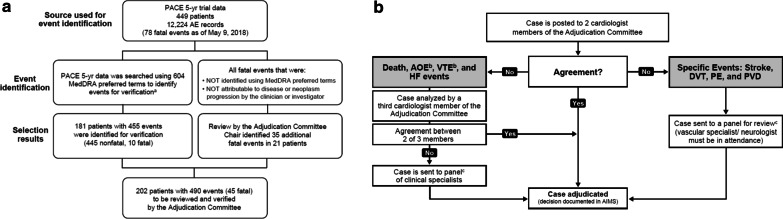
CONSORT flow diagram and process for adjudication of arterial occlusive events (AOEs). **A** CONSORT diagram: Identification of AOEs for review by the adjudication committee. **B** Adjudication process flow charts. *AE* adverse event, *AC* adjudication committee, *AIM* Applied Clinical Intelligence Information Management System, *MedDRA* Medical Dictionary for Regulatory Activities, *PACE* Ponatinib Ph+ ALL and CML Evaluation, *PE* pulmonary embolism, *PVD* peripheral vascular disease, *VTE* venous thromboembolism. ^a^The Adjudication Committee also reviewed any events included in the Cardiac Failure Standard MedDRA Query (SMQ) to determine whether any heart failure events were AOEs. ^b^AOEs evaluated on the left panel excluded events evaluated in the right panel (stroke, DVT, and PE). ^c^Per the charter, panel meetings were convened to discuss events for which a decision was not reached via independent voting. The quorum for panel meeting attendance was dependent on the type of event(s) to be discussed (i.e., cardiologist, neurologist, or vascular specialist)

To ensure all relevant potential events were captured, the PACE AE dataset (449 patients with 12,224 AE records; extraction date: May 9, 2018) was searched using a comprehensive set of 604 preferred terms potentially relevant to AOEs that was developed by the sponsor (Table 2). This search strategy, which was more comprehensive than that used in initial analyses of the PACE trial, identified 181 patients and 455 AE records for adjudication (Fig. 1A). In addition, all patient deaths not attributable to disease progression by the clinical investigator were reviewed by the chair of the adjudication committee (described below) for identification of potential fatal AOEs. The adjudication committee identified 45 fatal events for review. In total, 202 patients and 490 events were submitted to the independent adjudication committee for review (Fig. 1A).

**Table 2:** List of 604 preferred terms used to identify AEs for adjudication

Preferred term (MEdDRA 21.0)		
Acute aortic syndrome	Diplegia	Pituitary infarction
Acute coronary syndrome	Directional Doppler flow tests abnormal	Placental infarction
Acute myocardial infarction	Dissecting coronary artery aneurysm	Pneumatic compression therapy
Administration site thrombosis	Disseminated intravascular coagulation	Poor peripheral circulation
Adrenal thrombosis	Disseminated intravascular coagulation in newborn	Popliteal artery entrapment syndrome
Agnosia	Dry gangrene	Portal shunt procedure
Amaurosis	Dysarthria	Portal vein cavernous transformation
Amaurosis fugax	ECG electrically inactive area	Portal vein occlusion
Amputation	ECG signs of myocardial infarction	Portal vein stenosis
Angina pectoris	ECG signs of myocardial ischaemia	Portal vein thrombosis
Angina unstable	Electrocardiogram Q wave abnormal	Portosplenomesenteric venous thrombosis
Anginal equivalent	Electrocardiogram ST segment abnormal	Post angioplasty restenosis
Angiogram abnormal	Electrocardiogram ST segment depression	Post cardiac arrest syndrome
Angiogram cerebral abnormal	Electrocardiogram ST segment elevation	Post procedural myocardial infarction
Angiogram peripheral abnormal	Electrocardiogram ST-T segment abnormal	Post procedural pulmonary embolism
Angioplasty	Electrocardiogram ST-T segment depression	Post procedural stroke
Angiosclerosis	Electrocardiogram ST-T segment elevation	Post stroke depression
Anterior segment ischaemia	Electrocardiogram T wave abnormal	Post thrombotic syndrome
Aortic arteriosclerosis	Electrocardiogram T wave inversion	Posthaemorrhagic hydrocephalus
Aortic bypass	Electrocardiogram U wave inversion	Postinfarction angina
Aortic embolus	Embolia cutis medicamentosa	Postoperative thrombosis
Aortic occlusion	Embolic cerebral infarction	Postpartum thrombosis
Aortic restenosis	Embolic pneumonia	Postpartum venous thrombosis
Aortic stenosis	Embolic stroke	Precerebral arteriosclerosis
Aortic surgery	Embolism	Precerebral artery occlusion
Aortic thrombosis	Embolism arterial	Precerebral artery thrombosis
Aortogram abnormal	Embolism venous	Prinzmetal angina
Aphasia	Endarterectomy	Profundaplasty
Application site thrombosis	Exercise electrocardiogram abnormal	Prosthetic vessel implantation
Arm amputation	Exercise test abnormal	Pulmonary artery occlusion
Arterectomy	External counterpulsation	Pulmonary artery stenosis
Arterectomy with graft replacement	Extremity necrosis	Pulmonary artery therapeutic procedure
Arterial bypass occlusion	Extrinsic iliac vein compression	Pulmonary artery thrombosis
Arterial bypass operation	Femoral artery embolism	Pulmonary embolism
Arterial bypass stenosis	Finger amputation	Pulmonary endarterectomy
Arterial bypass thrombosis	Foetal cerebrovascular disorder	Pulmonary infarction
Arterial disorder	Foot amputation	Pulmonary microemboli
Arterial graft	Gangrene	Pulmonary thrombosis
Arterial insufficiency	Gastrointestinal ischaemia	Pulmonary tumour thrombotic microangiopathy
Arterial occlusive disease	Glomerular vascular disorder	Pulmonary vein occlusion
Arterial restenosis	Graft ischaemia	Pulmonary vein stenosis
Arterial stenosis	Graft thrombosis	Pulmonary veno-occlusive disease
Arterial stent insertion	Haemorrhage coronary artery	Pulmonary venous thrombosis
Arterial therapeutic procedure	Haemorrhagic adrenal infarction	Quadriparesis
Arterial thrombosis	Haemorrhagic cerebral infarction	Quadriplegia
Arteriogram abnormal	Haemorrhagic infarction	Raynaud's phenomenon
Arteriogram carotid abnormal	Haemorrhagic stroke	Renal arteriosclerosis
Arteriogram coronary abnormal	Haemorrhagic transformation stroke	Renal artery angioplasty
Arteriogram renal abnormal	Haemorrhagic vasculitis	Renal artery arteriosclerosis
Arteriosclerosis	Haemorrhoids thrombosed	Renal artery occlusion
Arteriosclerosis coronary artery	Hand amputation	Renal artery stenosis
Arteriosclerosis Monckeberg type	Hemianaesthesia	Renal artery thrombosis
Arteriosclerotic gangrene	Hemiparesis	Renal embolism
Arteriosclerotic retinopathy	Hemiplegia	Renal infarct
Arteriospasm coronary	Heparin-induced thrombocytopenia	Renal ischaemia
Arteriotomy	Hepatic artery embolism	Renal vascular thrombosis
Arteriovenous fistula occlusion	Hepatic artery occlusion	Renal vein embolism
Arteriovenous fistula thrombosis	Hepatic artery stenosis	Renal vein occlusion
Arteriovenous graft site stenosis	Hepatic artery thrombosis	Renal vein thrombosis
Arteriovenous graft thrombosis	Hepatic infarction	Retinal artery embolism
Arteritis	Hepatic ischaemia	Retinal artery occlusion
Artificial blood vessel occlusion	Hepatic vascular thrombosis	Retinal artery stenosis
Atherectomy	Hepatic vein embolism	Retinal artery thrombosis
Atherosclerotic plaque rupture	Hepatic vein occlusion	Retinal infarction
Atrial appendage closure	Hepatic vein stenosis	Retinal ischaemia
Atrial thrombosis	Hepatic vein thrombosis	Retinal vascular disorder
Axillary vein thrombosis	Homans' sign positive	Retinal vascular occlusion
Balint's syndrome	Hypothenar hammer syndrome	Retinal vascular thrombosis
Basal ganglia infarction	Hypoxic–ischaemic encephalopathy	Retinal vein occlusion
Basal ganglia stroke	Iliac artery disease	Retinal vein thrombosis
Basilar artery occlusion	Iliac artery embolism	Reversible cerebral vasoconstriction syndrome
Basilar artery stenosis	Iliac artery occlusion	Reversible ischaemic neurological deficit
Basilar artery thrombosis	Iliac vein occlusion	Right hemisphere deficit syndrome
Biliary ischaemia	Implant site thrombosis	Scan myocardial perfusion abnormal
Blindness transient	Incision site vessel occlusion	Shunt occlusion
Blood creatine phosphokinase abnormal	Infarction	Shunt thrombosis
Blood creatine phosphokinase increased	Inferior vena cava syndrome	SI QIII TIII pattern
Blood creatine phosphokinase MB abnormal	Inferior vena caval occlusion	Silent myocardial infarction
Blood creatine phosphokinase MB increased	Infusion site thrombosis	Skin ulcer
Bone infarction	Injection site thrombosis	Soft tissue necrosis
Bone marrow ischaemia	Inner ear infarction	Spinal artery embolism
Brachial artery entrapment syndrome	Instillation site thrombosis	Spinal artery thrombosis
Brachiocephalic arteriosclerosis	Intermittent claudication	Spinal cord infarction
Brachiocephalic artery occlusion	Interscapulothoracic amputation	Spinal cord ischaemia
Brachiocephalic artery stenosis	Intestinal infarction	Spinal vascular disorder
Brachiocephalic vein occlusion	Intestinal ischaemia	Splenic artery stenosis
Brachiocephalic vein stenosis	Intra-aortic balloon placement	Splenic artery thrombosis
Brachiocephalic vein thrombosis	Intracardiac mass	Splenic embolism
Brain hypoxia	Intracardiac thrombus	Splenic infarction
Brain stem embolism	Intracranial artery dissection	Splenic thrombosis
Brain stem infarction	Intracranial venous sinus thrombosis	Splenic vein occlusion
Brain stem ischaemia	Intraoperative cerebral artery occlusion	Splenic vein thrombosis
Brain stem stroke	Ischaemia	Spontaneous amputation
Brain stem thrombosis	Ischaemic cardiomyopathy	Stoma site thrombosis
Budd–Chiari syndrome	Ischaemic cerebral infarction	Stress cardiomyopathy
Capsular warning syndrome	Ischaemic contracture of the left ventricle	Stress echocardiogram abnormal
Cardiac arrest	Ischaemic enteritis	Stroke in evolution
Cardiac discomfort	Ischaemic gastritis	Subclavian artery embolism
Cardiac stress test abnormal	Ischaemic heart disease prophylaxis	Subclavian artery occlusion
Cardiac ventricular scarring	Ischaemic hepatitis	Subclavian artery stenosis
Cardiac ventricular thrombosis	Ischaemic limb pain	Subclavian artery thrombosis
Cardiopulmonary exercise test abnormal	Ischaemic mitral regurgitation	Subclavian coronary steal syndrome
Cardio-respiratory arrest	Ischaemic nephropathy	Subclavian steal syndrome
Cardiovascular disorder	Ischaemic neuropathy	Subclavian vein occlusion
Cardiovascular insufficiency	Ischaemic pancreatitis	Subclavian vein stenosis
Carotid angioplasty	Ischaemic skin ulcer	Subclavian vein thrombosis
Carotid arterial embolus	Ischaemic stroke	Subendocardial ischaemia
Carotid arteriosclerosis	Jugular vein occlusion	Superior mesenteric artery syndrome
Carotid artery bypass	Jugular vein thrombosis	Superior sagittal sinus thrombosis
Carotid artery calcification	Kounis syndrome	Superior vena cava occlusion
Carotid artery disease	Lacunar infarction	Superior vena cava syndrome
Carotid artery insufficiency	Lacunar stroke	Surgical vascular shunt
Carotid artery occlusion	Lateral medullary syndrome	Testicular infarction
Carotid artery restenosis	Leg amputation	Thalamic infarction
Carotid artery stenosis	Leriche syndrome	Thrombectomy
Carotid artery stent insertion	Limb amputation	Thromboangiitis obliterans
Carotid artery stent removal	Limb traumatic amputation	Thromboembolectomy
Carotid artery thrombosis	Macular ischaemia	Thrombolysis
Carotid endarterectomy	Mahler sign	Thrombophlebitis
Carotid revascularisation	May–Thurner syndrome	Thrombophlebitis migrans
Catheter site thrombosis	Medical device site thrombosis	Thrombophlebitis neonatal
Catheterisation venous	Mesenteric arterial occlusion	Thrombophlebitis superficial
Cavernous sinus thrombosis	Mesenteric arteriosclerosis	Thrombosed varicose vein
Central pain syndrome	Mesenteric artery embolism	Thrombosis
Central venous catheterisation	Mesenteric artery stenosis	Thrombosis corpora cavernosa
Cerebellar artery occlusion	Mesenteric artery stent insertion	Thrombosis in device
Cerebellar artery thrombosis	Mesenteric artery thrombosis	Thrombosis mesenteric vessel
Cerebellar embolism	Mesenteric phlebosclerosis	Thrombosis prophylaxis
Cerebellar infarction	Mesenteric vascular insufficiency	Thrombotic cerebral infarction
Cerebellar ischaemia	Mesenteric vascular occlusion	Thrombotic microangiopathy
Cerebellar stroke	Mesenteric vein thrombosis	Thrombotic stroke
Cerebral arteriosclerosis	Mesenteric venous occlusion	Thrombotic thrombocytopenic purpura
Cerebral artery embolism	Microembolism	Thyroid infarction
Cerebral artery occlusion	Microvascular coronary artery disease	Toe amputation
Cerebral artery restenosis	Migrainous infarction	Tongue infarction
Cerebral artery stenosis	Millard–Gubler syndrome	Transient ischaemic attack
Cerebral artery thrombosis	Monoparesis	Transverse sinus thrombosis
Cerebral autosomal dominant arteriopathy with subcortical infarcts and leukoencephalopathy	Monoplegia	Troponin I increased
Cerebral congestion	Moyamoya disease	Troponin increased
Cerebral gas embolism	Myocardial hypoxia	Troponin T increased
Cerebral hypoperfusion	Myocardial infarction	Truncus coeliacus thrombosis
Cerebral infarction	Myocardial ischaemia	Tumour embolism
Cerebral infarction foetal	Myocardial necrosis	Tumour thrombosis
Cerebral ischaemia	Myocardial necrosis marker increased	Ultrasonic angiogram abnormal
Cerebral microembolism	Myocardial reperfusion injury	Ultrasound Doppler abnormal
Cerebral reperfusion injury	Myocardial stunning	Umbilical cord occlusion
Cerebral revascularisation	Necrosis	Umbilical cord thrombosis
Cerebral septic infarct	Necrosis ischaemic	Uterine ischaemia
Cerebral small vessel ischaemic disease	Nephroangiosclerosis	Vaccination site thrombosis
Cerebral thrombosis	NIH stroke scale abnormal	Vascular access site occlusion
Cerebral vascular occlusion	NIH stroke scale score decreased	Vascular access site thrombosis
Cerebral vasoconstriction	NIH stroke scale score increased	Vascular encephalopathy
Cerebral venous thrombosis	Non-cardiac chest pain	Vascular graft
Cerebrospinal thrombotic tamponade	Obstetrical pulmonary embolism	Vascular graft occlusion
Cerebrovascular accident	Obstructive shock	Vascular graft restenosis
Cerebrovascular accident prophylaxis	Ocular ischaemic syndrome	Vascular graft stenosis
Cerebrovascular disorder	Ocular vascular disorder	Vascular graft thrombosis
Cerebrovascular insufficiency	Omental infarction	Vascular insufficiency
Cerebrovascular operation	Ophthalmic vein thrombosis	Vascular occlusion
Cerebrovascular stenosis	Optic ischaemic neuropathy	Vascular operation
Chest discomfort	Optic nerve infarction	Vascular pseudoaneurysm thrombosis
Chest pain	Ovarian vein thrombosis	Vascular shunt
Choroidal infarction	Paget–Schroetter syndrome	Vascular skin disorder
Choroidal sclerosis	Pancreatic infarction	Vascular stenosis
Claudication of jaw muscles	Papillary muscle infarction	Vascular stent insertion
Clumsiness	Paradoxical embolism	Vascular stent occlusion
Coeliac artery occlusion	Paralysis	Vascular stent restenosis
Coeliac artery stenosis	Paraneoplastic thrombosis	Vascular stent stenosis
Colitis ischaemic	Paraparesis	Vascular stent thrombosis
Collateral circulation	Paraplegia	Vasculitis
Compression garment application	Paresis	Vasoconstriction
Computerised tomogram coronary artery abnormal	Pelvic venous thrombosis	Vasodilation procedure
Coronary angioplasty	Penetrating atherosclerotic ulcer	Vena cava embolism
Coronary arterial stent insertion	Penile artery occlusion	Vena cava filter insertion
Coronary artery bypass	Penile vein thrombosis	Vena cava filter removal
Coronary artery compression	Percutaneous coronary intervention	Vena cava thrombosis
Coronary artery disease	Perinatal stroke	Venogram abnormal
Coronary artery dissection	Peripheral arterial occlusive disease	Venoocclusive disease
Coronary artery embolism	Peripheral arterial reocclusion	Venoocclusive liver disease
Coronary artery insufficiency	Peripheral artery angioplasty	Venous angioplasty
Coronary artery occlusion	Peripheral artery bypass	Venous occlusion
Coronary artery reocclusion	Peripheral artery occlusion	Venous operation
Coronary artery restenosis	Peripheral artery restenosis	Venous recanalisation
Coronary artery stenosis	Peripheral artery stenosis	Venous repair
Coronary artery surgery	Peripheral artery stent insertion	Venous stenosis
Coronary artery thrombosis	Peripheral artery thrombosis	Venous stent insertion
Coronary brachytherapy	Peripheral coldness	Venous thrombosis
Coronary bypass stenosis	Peripheral embolism	Venous thrombosis in pregnancy
Coronary bypass thrombosis	Peripheral endarterectomy	Venous thrombosis limb
Coronary endarterectomy	Peripheral ischaemia	Venous thrombosis neonatal
Coronary no-reflow phenomenon	Peripheral revascularisation	Vertebral artery occlusion
Coronary ostial stenosis	Peripheral vascular disorder	Vertebral artery stenosis
Coronary revascularisation	Periprocedural myocardial infarction	Vertebral artery thrombosis
Coronary vascular graft occlusion	Phlebectomy	Vertebrobasilar insufficiency
Coronary vascular graft stenosis	Phlebitis	Vessel puncture site occlusion
Coronary vein stenosis	Phlebosclerosis	Vessel puncture site thrombosis
Deep vein thrombosis		Vestibular ischaemia
Deep vein thrombosis postoperative		Visceral venous thrombosis
Delayed ischaemic neurological deficit		Visual acuity reduced transiently
Dependent rubor		Visual agnosia
Device embolisation		Visual midline shift syndrome
Device occlusion		Wall motion score index abnormal
Device related thrombosis		
Diabetic macroangiopathy		
Diabetic microangiopathy		
Diabetic vascular disorder

An individual case package containing all available clinical information (including medical history) was created for each event and provided to the adjudication committee members for their review. If a patient experienced more than 1 event within 48 h, these events were adjudicated as potentially representing a single clinical event, unless the case evidence suggested they were independent events. Individual events occurring > 48 h apart were adjudicated as independent events. All data were from the clinical trial database that was in SAS format and structured in conformance to CDISC SDTM format; no other source material was available.

#### Adjudication procedure

An adjudication committee of academic research clinicians who are highly experienced in adjudication activities in cardiovascular trials was appointed by ACI Clinical. The adjudication committee of 5 independent academic experts (3 cardiologists, 1 vascular medicine specialist, and 1 vascular neurologist) retrospectively adjudicated suspected cases of arterial occlusive events in the PACE study. The committee followed a predefined process outlined in the adjudication charter developed by ACI clinical. The charter defined the responsibilities of the adjudication committee and the adjudication endpoints using established definitions developed by the 2014 American College of Cardiology (ACC)/American Heart Association (AHA) guideline [10], and the definitions for cardiovascular and stroke outcomes developed by the Standardized Data Collection for Cardiovascular Trials Initiative (SCTI) and the US Food and Drug Administration [11, 12]. All suspected AOEs identified in the PT search were assessed using the charter definitions (Table 3) for myocardial infarction; heart failure if attributed to an AOE, which may include coronary artery disease, arterial hypertension, cardiomyopathy, or myocardial infarction; hospitalization for unstable angina; stroke and other cerebrovascular events; and peripheral vascular disease. Any events meeting the criteria of these endpoints were considered adjudicated AOEs. Specific criteria were required (e.g., revascularization, change in cardiac biomarkers, diagnostic evidence as shown by computerized tomography scan, magnetic resonance imaging, etc.) to determine the presence of a clinical endpoint. The adjudication committee members were blind to ponatinib dose at the time of the event, whether dose modifications were made, and the investigator’s opinion on AE causality.

**Table 3:** Adjudication committee prespecified definitions of events

Events	Definitions
Cardiovascular (CV) death	The cause of death will be determined by the principal condition that caused the death, not the immediate mode of death. Members of the adjudication committee will review all available information and use their clinical expertise to adjudicate the cause of death
	CV death includes death resulting from an acute myocardial infarction (MI), sudden cardiac death, death due to heart failure (HF), death due to stroke, death due to CV procedures, death due to CV hemorrhage, death due to pulmonary embolism, and death due to other CV causes
Death associated with acute myocardial infarction	Refers to a death by any CV mechanism (e.g., arrhythmia, sudden death, heart failure, stroke, pulmonary embolus, peripheral arterial disease) ≤ 30 days after a MI related to the immediate consequences of the MI, such as progressive heart failure or recalcitrant arrhythmia. Acute MI should be verified to the extent possible by the diagnostic criteria outlined for acute MI (see below) or by autopsy findings showing recent MI or recent coronary thrombosis
	Death resulting from a procedure to treat a MI (percutaneous coronary intervention (PCI), coronary artery bypass graft surgery (CABG), or to treat a complication resulting from MI, should also be considered death due to acute MI
	Death resulting from an elective coronary procedure to treat myocardial ischemia (i.e., chronic stable angina) or death due to a MI that occurs as a direct consequence of a CV investigation/procedure/operation should be considered as a death due to a CV procedure
Sudden cardiac death	Sudden cardiac death refers to death that occurs unexpectedly, not following an acute MI (as defined above) and includes the following deaths:
	Witnessed and occurring without new or worsening symptoms
	Witnessed within 60 min of the onset of new or worsening cardiac symptoms, unless the symptoms suggest acute MI
	Witnessed and attributed to an identified arrhythmia (e.g., captured on an electrocardiographic (ECG) recording or witnessed on a monitor, or unwitnessed but found on implantable cardioverter-defibrillator review)
	After unsuccessful resuscitation from cardiac arrest (e.g., implantable cardioverter-defibrillator [ICD] unresponsive sudden cardiac death, pulseless electrical activity arrest)
	After successful resuscitation from cardiac arrest and without identification of a specific cardiac or non-cardiac etiology
	Unwitnessed death in a subject seen alive and clinically stable ≤ 24 h prior to being found dead without any evidence supporting a specific non-CV cause of death (information regarding the patient’s clinical status preceding death should be provided, if available)
	Note: Unless additional information suggests an alternate specific cause of death (e.g., Death due to other CV causes), if a patient is seen alive ≤ 24 h of being found dead, sudden cardiac death should be recorded. For patients who were not observed alive within 24 h of death, undetermined cause of death should be recorded (e.g., a subject found dead in bed, but who had not been seen by family for several days)
	Note: Successful resuscitation without death should be captured as a resuscitated sudden cardiac death in the non-fatal voting flow
Death due to HF	Refers to death associated with clinically worsening symptoms and/or signs of HF regardless of etiology. Deaths due to HF can have various etiologies, including single or recurrent MIs, ischemic or non-ischemic cardiomyopathy, hypertension, or valvular disease
	Note: Due to the pro-thrombotic nature of the subject population, a thrombo-embolic option is included during voting. See rules in the non-fatal heart failure definition
Death due to stroke	Refers to death within 30 days that is either a direct consequence of the stroke or a complication of the stroke. Acute stroke should be verified to the extent possible by the diagnostic criteria outlined for stroke
Death due to CV procedures	Refers to death caused by the immediate complications of a cardiac procedure not in the context of treatment for acute MI
Death due to CV hemorrhage	Refers to death related to hemorrhage such as a non-stroke intracranial hemorrhage, non-procedural or non-traumatic vascular rupture (e.g., aortic aneurysm), or hemorrhage causing cardiac tamponade
Death due to other CV causes	Refers to a CV death not included in the above categories but with a specific, known cause (e.g., pulmonary embolism or peripheral vascular disease (venous or arterial disease)
Non-CV death	Non-CV death is defined as any death with a specific cause that is not thought to be of CV nature. Adjudication committee members will be asked to indicate the most likely cause of non-cardiovascular death on their voting form
	Examples of non-CV death are: pulmonary causes, renal causes, gastrointestinal causes, hepatobiliary causes, pancreatic causes, infection (including sepsis), inflammatory (e.g., systemic inflammatory response syndrome (SIRS))/immune (including autoimmune)(may include anaphylaxis from environmental (e.g., food allergies), hemorrhage that is neither cardiovascular bleeding or stroke, non-CV procedure or surgery, trauma, suicide, non-prescription drug reaction or overdose, prescription drug reaction or overdose (many include anaphylaxis), neurological (non-cardiovascular), malignancy (i.e., new malignancy, worsening of prior malignancy) or other (should be specified)
Undetermined cause of death	Undetermined cause of death refers to a death not attributable to one of the above categories. Inability to classify the cause of death may be due to lack of information (e.g., the only available information is “patient died”) or when there is insufficient supporting information or detail to assign the cause of death. In general, most deaths should be classifiable as CV or non-CV, and the use of this category of death, therefore, should be discouraged and should apply to few patients in well-run clinical trials
Non-fatal event definitions	
Myocardial infarction (non-fatal)	Criteria for acute MI: The term MI should be used when there is evidence of myocardial necrosis in a clinical setting consistent with acute myocardial ischemia. In general MI is defined as a combination of evidence of myocardial necrosis (changes in cardiac biomarkers) and supporting information (derived from the clinical presentation, electrocardiographic changes or the results of a myocardial or coronary artery imaging). Under these conditions, any one of the following criteria A to G meets the diagnosis for MI
	Spontaneous MI (type 1): To identify a type 1 MI, patients should demonstrate spontaneous symptoms of myocardial ischemia unprovoked by supply/demand inequity, together with at least one of the following criteria:
	Cardiac biomarker elevation: Troponin is the preferred marker for use to adjudicate the presence of acute MI. At least one value should show a rise and/or fall above the lowest cut-point providing 10% imprecision (typically the upper reference limit for the troponin run per standard of clinical care). Creatine kinase-MB is a secondary choice to troponin; a rise of CK-MB above the local upper reference limit would be consistent with myocardial injury. Total CK may be used in the absence of CK-MB and troponin
	Imaging evidence of new non-viable myocardium or new wall motion abnormality
	ECG changes consistent with new ischemic changes
	ECG changes indicative of new ischemia [new ST-T changes or new left bundle branch block (LBBB)]*
	Development of pathological Q-waves in the ECG**
	*ECG manifestations of acute myocardial ischemia (in absence of left ventricular hypertrophy (LVH) and left bundle branch block (LBBB)):
	ST elevation: New ST elevation at the J-point in two contiguous leads with the cut-off points: ≥ 0.2 mV in men or ≥ 0.15 mV in women in leads V2–V3 and/or ≥ 0.1 mV in other leads
	ST depression and T-wave changes: New horizontal or down- sloping ST depression ≥ 0.05 mV in two contiguous leads; and/or T inversion ≥ 0.1 mV in two contiguous leads with prominent R-wave or R/S ratio > 1
	**Pathological Q-waves:
	Any Q-wave in leads V2–V3 ≥ 0.02 s or QS complex in leads V2 and V3
	Q-wave ≥ 0.03 s and ≥ 0.1 mV deep or QS complex in leads I, II, aVL, aVF, or V4-V6 in any two leads of a contiguous lead grouping (I, aVL, V6; V4–V6; II, III, and aVF)
	“Demand” related MI (type 2): Patients with type 2 MI should be considered with similar diagnostic criteria as a type 1 MI, however type 2 MI should be considered present when myocardial ischemia and infarction are consequent to supply/demand inequity, rather than a spontaneous plaque rupture and coronary thrombosis
	Percutaneous coronary intervention-related MI (type 4a): For percutaneous coronary interventions (PCI) in patients with normal baseline troponin values, elevations of cardiac biomarkers above the 99th percentile URL, within 24 h of the procedure, are indicative of peri-procedural myocardial necrosis. By convention, increases of biomarkers greater than 5 × 99th percentile URL (Troponin or CK-MB > 5 × 99th percentile URL) are consistent with PCI-related MI. If the cardiac biomarker is elevated prior to PCI, a ≥ 20% increase of the value in the second cardiac biomarker sample within 24 h of the PCI and documentation that cardiac biomarker values were decreasing (2 samples at least 6 h apart) prior to the suspected recurrent MI is also consistent with PCI-related MI. In addition to biomarker elevation one of the following must exist:
	Symptoms suggestive of myocardial ischemia
	New ischemic ECG changes or new LBBB
	Angiographic findings consistent with procedural complication (e.g., Loss of patency, persistent slow/non-flow or embolization)
	Imaging demonstration of new loss of viable myocardium or new regional wall motion abnormality
	MI associated with stent thrombosis or stent restenosis as documented by angiography or at autopsy will also be captured as subtypes 4b and 4c
	Stent thrombosis related MI (type 4b): MI associated with stent thrombosis as detected by coronary angiography or at autopsy, where symptoms suggestive of myocardial ischemia are present, and with a rise and/or fall of cardiac biomarker values with at least 1 value > 99th percentile of the URL. If found with autopsy, it will be captured under cardiac death
	Definite stent thrombosis is considered to have occurred by either angiographic or pathological confirmation:
	Angiographic confirmation of stent thrombosis (Incidental angiographic documentation of stent occlusion in the absence of clinical signs or symptoms is not considered a confirmed stent thrombosis [silent occlusion]). The presence of a thrombus (intracoronary) that originates in the stent or in the segment 5 mm proximal or distal to the stent and presence of at least 1 of the following criteria within a 48-h time window:
	Acute onset of ischemic symptoms at rest
	New ischemic ECG changes that suggest acute ischemia
	Typical rise and fall in cardiac biomarkers (refer to definition of spontaneous MI)
	Non-occlusive thrombus
	Intracoronary thrombus is defined as a (spheric, ovoid, or irregular) non-calcified filling defect or lucency surrounded by contrast material (on 3 sides or within a coronary stenosis) seen in multiple projections, or persistence of contrast material within the lumen, or a visible embolization of intraluminal material downstream
	Occlusive thrombus TIMI 0 or TIMI 1 intrastent or proximal to a stent up to the most adjacent proximal side branch or main branch (if originates from the side branch)
	Pathological confirmation of stent thrombosis: Evidence of recent thrombus within the stent determined at autopsy or via examination of tissue retrieved following thrombectomy
	Probable stent thrombosis: Clinical definition of probable stent thrombosis is considered to have occurred after intracoronary stenting in the following cases:
	Any unexplained death within the first 30 days
	Irrespective of the time after the index procedure, any MI that is related to documented acute ischemia in the territory of the implanted stent without angiographic confirmation of stent thrombosis and in the absence of any other obvious cause
	Stent restenosis-related MI (type 4c): MI associated with stent restenosis as detected by coronary angiography or at autopsy, occurring > 48 h after index PCI without evidence of stent thrombosis but with symptoms suggestive of myocardial ischemia, and with elevation of cardiac biomarker values to > 99th percentile of the URL. This classification also requires the following:
	Does not meet criteria for any other classification of MI
	Presence of a ≥ 50% stenosis at the site of previous successful stent PCI or a complex lesion and no other significant obstructive CAD of greater severity following:
	Initially successful stent deployment
	OR
	Dilatation of a coronary artery stenosis with balloon angioplasty to < 50% stenosis
	If found with autopsy, it will be captured under cardiac death
	Coronary artery bypass grafting-related MI (type 5): MI associated with CABG is arbitrarily defined by elevation of cardiac biomarker values > 10 × 99th percentile URL in patients with normal baseline cardiac biomarker values (≤ 99th percentile URL). In addition to any one of the following:
	New pathological Q-waves or new LBBB
	Angiographic documented new graft or new native coronary artery occlusion
	Imaging evidence of new loss of viable myocardium or new regional wall motion abnormality
Heart failure event	A heart failure event includes hospitalization for heart failure and may include any urgent outpatient visits for heart failure. The date of this event will be the day of hospitalization of the patient (including any overnight stay at the emergency room or chest pain unit) or the day of visit to the urgent outpatient center. Due to the pro-thrombotic nature of the subject population, a thrombo-embolic option is included during voting
	The following rules may be applied to indicate if heart failure is attributed to an AOE/VTE:
	Heart failure may be attributed to an AOE/VTE if related to coronary artery disease, hypertension, cardiomyopathy or myocardial infarction
	The relationship of heart failure to an AOE/VTE may be excluded if the underlying cause of heart failure is heart valve disorders, congenital heart disorders or arrhythmias
Heart failure requiring hospitalization	Heart failure hospitalization is defined as an event that meets all the following criteria:
	Patient is admitted to the hospital with a primary diagnosis of HF
	Patient’s length of stay in hospital extends for at least 24 h (or a change in calendar date if the hospital admission and discharge times are unavailable)
	Patient exhibits documented new or worsening symptoms due to HF on presentation, including at least ONE of the following:
	Dyspnea
	Dyspnea with exertion
	Orthopnea
	Paroxysmal nocturnal dyspnea
	Decrease exercise tolerance
	Fatigue
	Other symptoms of worsened end-organ perfusion or volume overload
	Patient has objective evidence of new/worsening HF, consisting of at least TWO physical examination findings OR one physical examination finding and at least one laboratory criterion, including:
	Physical examination findings considered to be due to heart failure
	Peripheral edema
	Increasing abdominal distention or ascites (in the absence of primary hepatic disease)
	Pulmonary rales/crackles/crepitations
	Increased jugular venous pressure and/or hepatojugular reflux
	S^3^ gallop
	Clinically significant or rapid weight gain thought to be related to fluid retention
	Laboratory evidence of new or worsening HF, if obtained within 24 h of presentation, including:
	Increased b-type natriuretic peptide (BNP)/N-terminal proBNP (NT-proBNP) concentrations consistent with decompensation of heart failure (such as BNP > 500 pg/mL or NT-proBNP > 1800 pg/mL). In patients with chronically elevated natriuretic peptides, a significant increase should be noted above baseline
	Radiological evidence of pulmonary congestion
	New or worsened bilateral pleural effusions
	Noninvasive diagnostic evidence of clinically significant elevated left or right-sided ventricular filling pressure or low cardiac input
	Invasive diagnostic evidence with right heart catheterization showing a pulmonary capillary wedge pressure (pulmonary artery occlusion pressure) ≥ 18 mmHg, central venous pressure ≥ 12 mmHg, or a cardiac index < 2.2 L/min/m^2^
	Patient receives initiation or intensification of treatment specifically for HF (at least one of the following):
	Augmentation in oral diuretic therapy or ACE inhibitor
	Intravenous diuretic or vasoactive agent (e.g., inotrope, vasopressor, or vasodilator)
	Mechanical or surgical intervention:
	Mechanical circulatory support (e.g., intra-aortic balloon pump, ventricular assist device, extracorporeal membrane oxygenation, total artificial heart)
	Mechanical fluid removal (e.g., dialysis, ultrafiltration, hemofiltration)
Urgent heart failure visit	An urgent heart failure visit is defined as an event that meets all the following criteria:
	The patient has an urgent, unscheduled office/practice or emergency department visit for a primary diagnosis of heart failure, but not meeting the criteria for a heart failure hospitalization
	All signs/symptoms for heart failure hospitalization (i.e., symptoms, physical examination findings/lab evidence of new or worsening HF as indicated under definition for Heart Failure Hospitalization) must be met
	The patient receives initiation or intensification of treatment specifically for heart failure, as detailed in the heart failure hospitalization section with the exception of oral diuretic therapy (which will not be sufficient)
Hospitalization for unstable angina	The date of this event will be the day of hospitalization of the patient including any overnight stay at an emergency room or chest pain unit
	Hospitalization for unstable angina is defined as an event that meets all the following criteria:
	Negative cardiac biomarkers and no evidence of acute MI
	Ischemic discomfort (angina or other symptoms thought to be equivalent) ≥ 10 min in duration occurring at rest or in an accelerating pattern with frequent episodes associated with progressively decreased exercise capacity
	Unscheduled hospitalization within 24 h of the most recent symptoms. Hospitalization is defined as an admission to an inpatient unit or a visit to an emergency department that results in at least a 24 h stay (or a change in calendar date if the hospital admission or discharge times are not available)
	At least one of the following:
	New or worsening ST or T-wave changes on resting ECG (in absence of confounders such as LBBB or LVH)
	ST Elevation: New transient (duration < 20 min) at the J point in two contiguous leads with the cut-points: ≥ 0.1 mV in all leads other than leads V2-V3 where the following cut-points apply: ≥ 0.2 mV in men ≥ 40 years (≥ 0.25 mV in men < 40 years) or ≥ 0.15 mV in women
	ST depression and T-wave changes: New horizontal or down-sloping ST depression ≥ 0.05 mV in two contiguous leads and/or a new T inversion ≥ 0.3 mV in two contiguous leads with prominent R -wave or R/S ratio > 1
	Definite evidence of inducible myocardial ischemia as demonstrated by one of the following and believed to be responsible for symptoms:
	Early positive stress test (defined as ST elevation or ≥ 2 mm ST depression prior to 5 mets)
	Stress echocardiography (reversible wall motion abnormality)
	Myocardial scintigraphy (reversible perfusion defect)
	MRI (myocardial perfusion deficit under pharmacologic stress)
	Angiographic evidence of new or worse ≥ 70% lesion (≥ 50% for left main lesion) and/or thrombus in an epicardial coronary artery that is believed to be responsible for the myocardial ischemic symptoms/signs
	Need for coronary revascularization procedure (PCI or CABG) for the presumed culprit lesion(s). This criterion would be fulfilled if revascularization was undertaken during the unscheduled hospitalization, or subsequent to transfer to another institution without interceding home discharge
Stroke	Stroke is defined as an acute episode of focal or global neurological dysfunction caused by brain, spinal cord, or retinal vascular injury as a result of hemorrhage or infarction. Strokes will be classified as ischemic, hemorrhagic, retinal artery occlusion or thrombosis or undetermined
	General
	Stroke is defined as an acute episode of focal or global neurological dysfunction caused by brain, spinal cord, or retinal vascular injury as a result of hemorrhage or infarction, with symptom duration of 24 h or more. Episodes lasting less than 24 h can be considered a stroke if there is an intervention to abort the stroke (e.g., thrombolytic therapy), diagnostic confirmation of the stroke, or patient death prior to reaching the 24 h duration
	Subdural and epidural hematomas are intracranial hemorrhagic events and are not strokes
	Diagnosis of stroke
	For the diagnosis of stroke, the following 4 criteria should be fulfilled:
	Acute onset* of a focal/global neurological deficit with at least one of the following:
	Change in level of consciousness
	Hemiplegia
	Hemiparesis
	Numbness or sensory loss affecting one side of the body
	Dysphasia/Aphasia
	Hemianopia (loss of half of the field of vision of one or both eyes)
	Other new neurological sign(s)/symptom(s) consistent with stroke
	*If the mode of onset is uncertain, a diagnosis of stroke may be made provided that there is no plausible non-stroke cause for the clinical presentation
	Duration of a focal/global neurological deficit ≥ 24 h
	OR
	< 24 h if
	This is because of at least one of the following therapeutic interventions:
	Pharmacologic (i.e., thrombolytic drug administration)
	Non-pharmacologic (i.e., neurointerventional procedure (e.g., intracranial angioplasty))
	or
	available brain imaging clearly documents a new hemorrhage or infarct
	or
	the neurological deficit results in death
	No other readily identifiable non-stroke cause for the clinical presentation (e.g., brain tumor, trauma, infection, hypoglycemia, peripheral lesion)
	Confirmation of the diagnosis by at least one of the following:**
	Neurology or neurosurgical specialist
	Brain imaging procedure (at least one of the following):
	CT scan
	MRI scan
	Cerebral vessel angiography
	Lumbar puncture (i.e., spinal fluid analysis diagnostic of subarachnoid hemorrhage)
	**If a stroke is reported but evidence of confirmation of the diagnosis by the methods outlined above is absent, the event will be discussed at a full EAC meeting. In such cases, the event may be adjudicated as a stroke on the basis of the clinical presentation alone, but full EAC consensus will be mandatory
	Classification of stroke
	Strokes are sub-classified as follows:
	Ischemic (non-hemorrhagic)
	Ischemic stroke is defined as an acute episode of focal cerebral, spinal or retinal dysfunction caused by infarction of central nervous system tissue. Hemorrhage may be a consequence of ischemic stroke. In this situation, the stroke is an ischemic stroke with hemorrhagic transformation and not a hemorrhagic stroke
	Hemorrhagic
	Hemorrhagic stroke is defined as an acute episode of focal or global cerebral or spinal dysfunction caused by intraparenchymal, intraventricular, or subarachnoid hemorrhage
	Retinal artery occlusion or thrombosis
	Retinal artery occlusion or thrombosis is defined as a blockage in one of the retinal arteries
	Occlusions may be caused by a thromboembolism or other risk factors such as atherosclerosis and arrhythmias
	Note: Amaurosis fugax is not considered part of this endpoint
	Undetermined stroke
	Undetermined stroke is defined as an acute episode of focal or global neurological dysfunction caused by presumed brain, spinal cord, as a result of hemorrhage or infarction but with insufficient information to allow categorization as #1 and #2 above
	Note: Given the scope of this study, stroke disability will not be measured. TIA definition was intentionally left out for this study; suspected TIA events will be identified for adjudication in order to rule out stroke
Venous thrombosis	Superficial vein thrombosis
	Superficial vein thrombosis (SVT) refers to a blood clot in one of the superficial veins near the surface of the body. There is usually an inflammatory reaction around the vein and may present with as a painful induration with erythema. An SVT can lead to a serious complication such as a higher risk for pulmonary embolism
	Superficial vein thrombosis could be documented by one of the following:
	Clinical symptoms (such as warmth, edema, ‘cord-like’ palpable mass, erythema, pain)
	Duplex ultrasound
Deep vein thrombosis	Deep vein thrombosis (DVT) refers to a blood clot in one of the deep veins (to include distal and proximal DVT). It may occur anywhere in the body but is most common in the extremities, a clot blocks blood circulation through these veins, which carry blood back to the heart. This commonly causes pain and swelling distal to the thrombus. Severe complications of DVT may occur when a clot embolizes to the lung
	Deep vein thrombosis could be documented by one of the following:
	Venous ultrasonography
	Compression ultrasonography (CUS)
	Impedance plethysmography (IPG)
	Venography
	CT scan
	MRI
	At autopsy
	Location
	Venous thrombosis (DVT and SVT) will be categorized for location by the EAC
	Members as follows:
	Lower limb
	Upper limb
	Retinal vein
	Abdominal viscera
	Other (e.g., more unusual sites of cerebral venous thrombosis)
Pulmonary embolism	A pulmonary embolism (PE) is a blood clot in the arteries of the lung that typically arise from the veins. The embolus not only prevents the exchange of oxygen and carbon dioxide via the lungs, but it also decreases blood supply to the lung tissue itself, potentially causing infarction. The most common symptoms include pleuritic chest pain, dyspnea, and hemoptysis. A PE may lead to sudden death. Death due to PE refers to death that is either a direct consequence or complication of a PE. Fatal PE is captured in the fatal definition section as death due to other CV causes
	Pulmonary embolism should be documented by supporting evidence found within any one of the following:
	CT scan
	Pulmonary angiogram
	Ventilation/perfusion lung scan (VPLS)
	Inconclusive spiral CT, pulmonary angiography or lung scintigraphy with demonstration of DVT in the lower extremities by CUS or venography with clinical, lab and EKG findings consistent with PE
	At autopsy
Other AOE/VTE	Peripheral vascular disease (PVD)
	Peripheral vascular disease refers to a blood circulation disorder outside of the heart and brain that causes the blood vessels to block, narrow or spasm. PVD can be either in veins or arteries. Physical symptoms may include weak pulses, wounds/ulcers that won’t heal, thin or pale skin
	PVD could be documented by one of the following:
	Doppler ultrasound
	Ankle-brachial index
	Angiography
	Magnetic resonance angiography
	Computerized tomography angiography
	Members will be asked to choose if this is a venous or arterial occlusive event
Revascularization procedures	For fatal and non-fatal cardiovascular endpoint events, members must also indicate if the event is associated with a revascularization procedure (PCI, CABG or PVI)
	Percutaneous coronary intervention (PCI)
	Defined as the placement of an angioplasty guidewire, balloon, or other device (e.g., stent, atherectomy, brachytherapy or thrombectomy catheter) into a native coronary artery or CABG for the purpose of mechanical coronary revascularization. The assessment of coronary lesion severity by intravascular ultrasonography, coronary flow reserve, or fractional flow reserve is not considered a PCI procedure
	Coronary artery bypass graft (CABG)
	Defined as a procedure performed to bypass partially or completely occluded coronary arteries with veins and/or arteries harvested from elsewhere in the body, thereby improving the blood supply to the coronary circulation supplying the myocardium
	Peripheral vascular intervention (PVI)
	Peripheral vascular intervention is a catheter-based or open surgical procedure designed to improve arterial or venous blood flow or otherwise modify or revise vascular conduits. Procedures may include, but are not limited to percutaneous transluminal balloon angioplasty, stent placement, thrombectomy, embolectomy, atherectomy, dissection repair, aneurysm exclusion, treatment of dialysis conduits, placement of various devices, intravascular thrombolysis or other pharmacotherapies, and open surgical bypass or revision

During the adjudication process, the committee reviewed all potential AOEs, as well as any AEs identified in a Cardiac Failure Standard MedDRA Query (SMQ), to determine whether any heart failure events were AOEs. Two members of the adjudication committee independently evaluated whether an individual case met the prespecified event definitions (Fig. 1B). If agreement between 2 members was not reached for cases of AOEs or heart failure, the case was reviewed by a third cardiologist adjudication committee member; if agreement was not reached with 3 votes, the case was reviewed at a panel meeting. If agreement was not reached for cases of stroke, deep vein thrombosis, pulmonary embolism, and peripheral vascular disease, the case was discussed at a panel meeting with the appropriate neurologist and/or vascular specialist member(s). All fatal events were decided by consensus of adjudicators.

Events that met one of the charter-defined endpoint definitions were further categorized depending on the event type (e.g., myocardial infarction, peripheral arterial occlusive disease, deep vein thrombosis, etc.). Non-adjudicated AOEs that were recorded as symptoms (e.g., "non-cardiac chest pain" or "claudication") with a low severity level and no accompanying changes in medication or hospitalization were adjudicated to not be AOEs unless they had an anatomic diagnosis provided (e.g., "severe superficial femoral artery stenosis"). If the term "infarction" was provided for stroke events, the adjudicators categorized the event as ischemic stroke. Revascularization was not always clearly reported by investigators.

### Statistics

Exposure-adjusted AOE rates were calculated as: (number of first events in interval)/(total exposure for interval in patient-years) × 100. The relative risk of serious AOEs was analyzed by baseline risk category in patients from the safety population for whom all baseline risk categories were available. Risk categories included commonly recognized cardiovascular risk factors for which data were collected (arterial hypertension, hypercholesterolemia, diabetes mellitus, and obesity), and history of heart disease (non-ischemic or ischemic).

## Results

### Patient disposition and baseline characteristics

Patient disposition and baseline characteristics in the PACE trial have been published [2, 3]. A total of 449 patients, including 270 CP-CML patients, 85 accelerated-phase (AP) CML patients, 62 blast-phase (BP) CML patients, and 32 Ph+ ALL patients, were enrolled between September 2010 and October 2011. Baseline characteristics are summarized in Table 4. Among all 449 patients, the median age was 59 years and 53% of patients were male. Most (93%) patients had received 2 or more prior TKIs. At baseline, 53% of patients had arterial hypertension, 49% had hypercholesterolemia, and 24% had BMI ≥ 30 kg/m^2^. Forty-three percent of patients had a baseline history of non-ischemic cardiac disease, and 23% had a history of ischemic cardiovascular disease. Safety data reviewed by the adjudication committee reflect data collected as of February 6, 2017, with median follow-up of 37.3 months for all patients and 56.8 months (range 0.1–73.1 months) for CP-CML patients.

**Table 4:** Baseline characteristics and disposition at end-of-study^3^

	CP-CML *n* = 270	Total *N* = 449
*Characteristic at baseline*		
Median age (range), y	60 (18–94)	59 (18–94)
Female, *n* (%)	126 (47)	211 (47)
Previous use of approved TKIs, *n* (%)^a^		
≥ 2 drugs	251 (93)	417 (93)
≥ 3 drugs	154 (57)	250 (56)
Median duration of previous treatment with approved TKIs (range), y^a^	5.4 (0.4–13.3)	4.6 (0.1–13.3)
Resistant or intolerant to dasatinib or nilotinib, *n* (%)		
Resistant	215 (80)	375 (84)
Intolerant only	39 (14)	49 (11)
Both resistant and intolerant	52 (19)	81 (18)
Mutation status, *n* (%)^b^		
No mutation detected	138 (51)	198 (44)
BCR::ABL1^T315I^	64 (24)	128 (29)
Best response of MMR or better to most recent regimen containing dasatinib or nilotinib, *n* (%)^c^	8 (3)	16 (4)
Baseline cardiovascular risk factors^d^		
Arterial hypertension	NA	240 (53)
Hypercholesterolemia	NA	219 (49)
Obesity	NA	109 (24)
Diabetes mellitus	NA	72 (16)
Baseline history of cardiovascular disease		
Non-ischemic cardiac disease	NA	193 (43)
Ischemic disease	NA	102 (23)
*Patient disposition at end of study*		
Median duration of treatment, mo (range)	32.1 (0.1–73.0)	16.7 (0.03–73.0)
Median follow-up, mo (range)	56.8 (0.1–73.1)	37.3 (0.1–73.1)
Median dose intensity, mg/d (range)	27.2 (5–45)	ND
Primary reason for discontinuation, *n* (%)		
Disease progression	29 (11)	105 (23)
Adverse event	57 (21)	79 (18)
Patient request	31 (11)	42 (9)
Lack of efficacy	15 (6)	26 (6)
Death^e^	9 (3)	26 (6)
Investigator decision	11 (4)	17 (4)
Lost to follow-up	0	3 (< 1)
Non-compliance	3 (1)	4 (< 1)
Protocol violation	2 (< 1)	2 (< 1)
Study closure^f^	90 (33)	107 (24)
Other^f,g^	14 (5)	28 (6)

*CML* chronic myeloid leukemia, *CP* chronic phase, *MMR* major molecular response, *ND* not determined, *TKI* tyrosine kinase inhibitor

^a^Approved TKIs were imatinib, nilotinib, dasatinib, and bosutinib. Previous investigational TKIs received by at least 1% of patients included radotinib (received by 2% of patients), bafetinib (2%), rebastinib (2%), and XL-228 (2%)

^b^Assessed by conventional Sanger sequencing at baseline

^c^Percentages were calculated according to the number of patients who received previous dasatinib or nilotinib: 256 patients with CP-CML, 80 patients with AP-CML, 61 patients with BP-CML, and 30 patients with Ph+ ALL

^d^Smoking and family history were not collected as part of the trial. Patients with significant or active cardiovascular disease, including myocardial infarction, unstable angina or congestive heart failure (in prior 3 months), or history of clinically significant atrial or ventricular arrhythmia were excluded from the trial

^e^Seven deaths were assessed by investigators as possibly or probably related to ponatinib (CP-CML: pneumonia, acute myocardial infarction; AP-CML: fungal pneumonia, gastrointestinal hemorrhage; BP-CML: hemorrhagic gastritis; Ph+ ALL: cardiac arrest, mesenteric arterial occlusion)

^f^Patients who continued to derive clinical benefit from their treatment had the option to receive ponatinib through alternative mechanisms

^g^This category includes stem cell transplantation (in 11 patients with CP-CML, 5 with AP-CML, 6 with BP-CML, and 1 with Ph+ ALL). The 9 CP-CML patients and 1 AP-CML patient who remained on study at the time of last response assessment are not included in this category.^3^

### Adjudication results

**Fig. 2:**
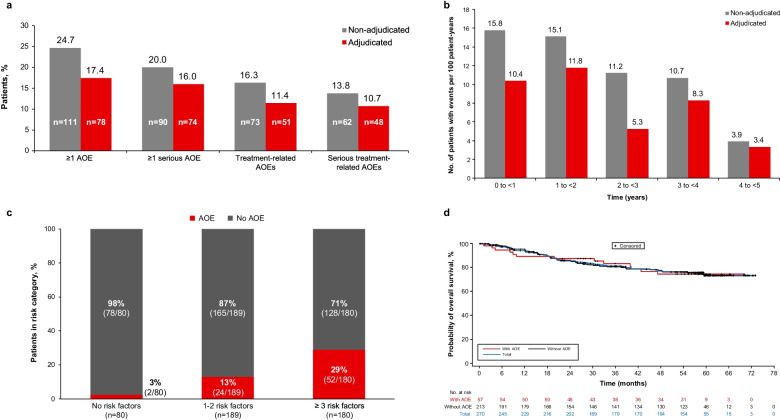
Arterial occlusive event (AOE) rates with ponatinib. **A** Rates of non-adjudicated and adjudicated AOEs. **B** Exposure-adjusted incidence of newly occurring arterial occlusive events (AOEs) by year (all patients). Later intervals excluded patients with prior events. Non-adjudicated values were published previously [3]. **C** Incidence of AOEs (adjudicated) by number of baseline risk factors (all patients). Risk factors included arterial hypertension, hypercholesterolemia, obesity, diabetes mellitus, non-ischemic cardiac disease, and ischemic disease. **D** Overall survival (OS) in chronic-phase chronic myeloid leukemia (CP-CML) patients with and without AOEs

Rates of adjudicated AOEs were lower than rates of non-adjudicated AOEs (Fig. 2A). Overall, 17% (78/449) of patients had adjudicated AOEs compared with 25% (111/449) with non-adjudicated AOEs. Most patients with serious AOEs were adjudicated as having serious AOEs (20% [90/449] non-adjudicated vs. 16% [74/449] adjudicated). Most (95% [74/78]) patients with adjudicated AOEs had serious AOEs. In CP-CML patients, rates of adjudicated AOEs (21% [57/270]) were also lower than rates of non-adjudicated AOEs (31% [84/270]); 95% [54/57] of CP-CML patients with adjudicated AOEs had serious AOEs. The rates of AOEs by AOE type (i.e., cardiovascular, cerebrovascular, and peripheral vascular) are presented for all patients in Table 5 and for CP-CML patients in Table 6.

**Table 5:** Rates of non-adjudicated and adjudicated AOEs by type in the total population (*n* = 449)

AOE	Non-adjudicated events^a^		Adjudicated events^b^	
	Any	Serious	Any	Serious
Any,	111 (25)	90 (20)	78 (17)	74 (16)
Cardiovascular^c^	59 (13)	44 (10)	38 (8)	37 (8)
Cardiovascular AOEs in ≥ 1% of patients				
Angina pectoris	28 (6)	15 (3)	0	0
Acute MI^d^	18 (4)	18 (4)	8 (2)	8 (2)
MI	^d^	^d^	10 (2)	10 (2)
Coronary artery disease	14 (3)	12 (3)	7 (2)	7 (2)
Acute coronary syndrome	7 (2)	7 (2)	7 (2)	7 (2)
Coronary artery occlusion	5 (1)	4 (1)	0	0
Cerebrovascular	41 (9)	33 (7)	28 (6)	25 (6)
Cerebrovascular AOEs in ≥ 1% of patients				
Cerebrovascular accident	11 (2)	11 (2)	7 (2)	7 (2)
Cerebral infarction	8 (2)	8 (2)	8 (2)	8 (2)
Carotid artery stenosis	7 (2)	6 (1)	7 (2)	5 (1)
Transient ischemic attack	6 (1)	4 (1)	0	0
Peripheral vascular	48 (11)	38 (8)	42 (9)	34 (8)
Peripheral vascular AOEs in ≥ 1% of patients				
Peripheral arterial occlusive disease	22 (5)	17 (4)	19 (4)	16 (4)
Intermittent claudication	11 (2)	1 (< 1)	0	0
Peripheral artery stenosis	10 (2)	8 (2)	8 (2)	7 (2)
Peripheral artery occlusion	7 (2)	5 (1)	7 (2)	5 (1)
Peripheral ischemia	7 (2)	4 (1)	5 (1)	0
Peripheral vascular disorder	5 (1)	4 (1)	0	0
Exposure-adjusted newly occurring AOEs, patients with events per 100 patient-years	13.8	10.6	8.9	8.4

Data are no. (%) of patients, unless otherwise specified

*AOE* arterial occlusive event, *CP-CML* chronic-phase chronic myeloid leukemia, *MedDRA* Medical Dictionary for Regulatory Activities, *MI* myocardial infarction, *PT* preferred term

^a^Categorization of AOEs is based on > 400 MedDRA preferred terms related to vascular ischemia or thrombosis

^b^Events that were adjudicated as an AOE by the adjudication committee

^c^Does not include arterial hypertension AEs

^d^Acute MI and MI were grouped as a single category in the non-adjudicated analysis

**Table 6:** Rates of AOEs non-adjudicated and adjudicated AOEs in CP-CML patients (*n* = 270)

AOE	Non-adjudicated events^a^		Adjudicated events^b^	
	Any	Serious	Any	Serious
Any,	84 (31)	69 (26)	57 (21)	54 (20)
Cardiovascular^c^	42 (16)	33 (12)	26 (10)	25 (9)
Cerebrovascular	35 (13)	28 (10)	25 (9)	22 (8)
Peripheral vascular	38 (14)	31 (11)	31 (11)	26 (10)
Exposure-adjusted newly occurring AOEs, patients with events per 100 patient-years	11.3	9.3	8.7	8.1

Data are no. (%) of patients, unless otherwise specified

*AOE* arterial occlusive event, *CP-CML* chronic-phase chronic myeloid leukemia, *MedDRA* Medical Dictionary for Regulatory Activities, *MI* myocardial infarction, *PT* preferred term

^a^Categorization of AOEs is based on > 400 MedDRA preferred terms related to vascular ischemia or thrombosis

^b^Events that were adjudicated as an AOE by the adjudication committee

^c^Does not include arterial hypertension AEs

The most common non-adjudicated and adjudicated AOEs and serious AOEs are summarized in Table 7. The most common (> 2%) non-adjudicated AOEs were angina pectoris (6%; 28/449), peripheral arterial occlusive disease (5%; 22/449), MI (4%; 18/449), coronary artery disease (3% [14/449]). The only adjudicated AOE reported in > 2% of patients was peripheral arterial occlusive disease (4% [16/449]). Non-adjudicated AOEs that were most commonly adjudicated as not AOEs were angina pectoris, non-cardiac chest pain, and chest pain, as these events were often recorded as symptoms (e.g., "non-cardiac chest pain" or "claudication") or presumptive diagnoses with a low severity level and no accompanying changes in medication or hospitalization.

**Table 7:** Arterial occlusive events (AOEs) in ≥ 2.0% of patients (*n* = 449)

AOE	Any AOE		Serious AOE	
	Non-adjudicated^a^	Adjudicated^b^	Non-adjudicated^a^	Adjudicated^b^
Angina pectoris	28 (6)	0	15 (3)	0
Peripheral arterial occlusive disease	22 (5)	19 (4)	17 (4)	16 (4)
Myocardial infarction	18 (4)	10 (2)	18 (4)	10 (2)
Coronary artery disease	14 (3)	7 (2)	12 (3)	7 (2)
Cerebrovascular accident	11 (2)	7 (2)	11 (2)	7 (2)
Intermittent claudication	11 (2)	0	1 (< 1)	0
Peripheral artery stenosis	10 (2)	8 (2)	8 (2)	7 (2)
Cerebral infarction	8 (2)	8 (2)	8 (2)	8 (2)
Acute coronary syndrome	7 (2)	7 (2)	7 (2)	7 (2)
Carotid artery stenosis	7 (2)	7 (2)	6 (1)	5 (1)
Peripheral artery occlusion	7 (2)	7 (2)	5 (1)	5 (1)
Peripheral ischemia	7 (2)	5 (1)	4 (1)	0

Data are no. (%) of patients

*MedDRA* Medical Dictionary for Regulatory Activities

^a^Categorization of AOEs is based on MedDRA preferred terms related to vascular ischemia or thrombosis

^b^Events adjudicated as AOEs by the cardiovascular endpoint Adjudication Committee

The exposure-adjusted incidence of adjudicated AOEs (8.9 patients with events per 100 patient-years) and serious AOEs (8.4 patients with events per 100 patient-years) was lower than the exposure-adjusted incidence of non-adjudicated AOEs (11.3 and 9.2 per 100 patient-years, respectively). The exposure-adjusted incidence of newly occurring AOEs decreased over time (Fig. 2B). The median time to onset of the first adjudicated AOE was 14.1 months (range: 0.1 to 49.5; Table 8).

**Table 8:** Time to onset of adjudicated AOEs

	Median time to first AOE (range), months	
	CP-CML patients	All patients
Any AOE	(*n* = 57) 16.3 (0.4, 49.5)	(*n* = 78) 14.1 (0.1, 49.5)
Cardiovascular AOE	(*n* = 26) 14.1 (0.6, 52.9)	(*n* = 38) 12.3 (0.3, 52.9)
Cerebrovascular AOE	(*n* = 25) 23.0 (0.4, 53.5)	(*n* = 28) 18.9 (0.4, 53.5)
Peripheral vascular AOE	(*n* = 31) 24.6 (1.8, 49.5)	(*n* = 42) 22.2 (0.1, 49.5)

### Resolution of AOEs, dose modifications, and discontinuations

Among the 78 patients with an adjudicated AOE, events resolved in 51 patients. Among 43 patients with just one AOE, 74% (32/43) had resolution of the event; 35 patients had multiple AOEs recorded, with 54% (19/35) patients having resolution of all the events. Most patients continued ponatinib after the AOE, including 36 patients (46%) who continued ponatinib without dose modification and 27 patients (35%) who had their doses reduced and/or interrupted after the event (Table 9). Seven patients (9%) discontinued ponatinib due to an adjudicated AOE. Rates of dose modifications following AOEs are summarized in Table 9.

**Table 9:** Ponatinib dose modifications following non-adjudicated and adjudicated arterial occlusive events (AOEs)^a^

	Any AOE		Serious AOE	
	Non-adjudicated^b^ (*n* = 111)	Adjudicated^c^ (*n* = 78)	Non-adjudicated^b^ (*n* = 90)	Adjudicated^c^ (*n* = 74)
No dose modification	46 (41)	36 (46)	28 (31)	31 (42)
Drug interrupted only	37 (33)	25 (32)	37 (41)	26 (35)
Dose reduced only	6 (5)	0	5 (6)	0
Dose reduced + drug interrupted	5 (5)	2 (3)	4 (4)	2 (3)
Drug interrupted + drug withdrawn	0	2 (3)	0	2 (3)
Drug withdrawn	17 (15)	5 (6)	16 (18)	5 (7)
Not applicable/unknown	0	8 (10)	0	8 (11)

Data are no. (%) of patients with an AOE

*MedDRA* Medical Dictionary for Regulatory Activities

^a^When a patient had multiple events, dose modification was derived as the most severe one across all events with the following severity order (high to low): drug withdrawn, drug reduced plus drug interrupted, drug reduced only, drug interrupted only, no dose modification

^b^Categorization of AOEs is based on MedDRA preferred terms related to vascular ischemia or thrombosis

^c^Events adjudicated as AOEs by the cardiovascular endpoint Adjudication Committee

### Risk factor analysis

The most common baseline risk factors in patients who developed an AOE were arterial hypertension and hypercholesterolemia (Table 10). Patients with adjudicated AOEs also had higher rates of concomitant use of antihypertensive medications, platelet aggregation inhibitor medications, and anti-diabetic agents compared with patients who did not have AOEs (Table 11).

**Table 10:** Prevalence of baseline risk factors by adjudicated AOE and serious AOE status

No. (%) of patients	Any AOE		Any serious AOE	
	No (*n* = 371)	Yes (*n* = 78)	No (*n* = 375)	Yes (*n* = 74)
Age, ≥ 65 years	118 (32)	37 (47)	120 (32)	35 (47)
Sex, male	187 (50)	51 (65)	188 (50)	50 (68)
History of ischemic disease	45 (12)	22 (28)	45 (12)	22 (30)
Diabetes mellitus	45 (12)	27 (35)	48 (13)	24 (32)
Baseline glucose grade ≥ 2	24 (6)	14 (18)	25 (7)	13 (18)
Venous thromboembolism	30 (8)	8 (10)	30 (8)	8 (11)
Arterial hypertension	181 (49)	59 (76)	185 (49)	55 (74.3)
Baseline blood pressure grade ≥ 2	32 (9)	7 (9)	32 (9)	7 (9)
Hypercholesterolemia	167 (45)	52 (67)	169 (45)	50 (68)
Baseline triglycerides grade ≥ 1	112 (30)	28 (36)	114 (30)	26 (35)
History of non-ischemic cardiac disease	120 (32)	30 (38)	121 (32)	29 (39)
Obesity	88 (24)	21 (27)	90 (24)	19 (26)
Baseline BMI ≥ 30 kg/m^−2^	86 (23)	21 (27)	88 (23)	19 (26)

*AOE* arterial occlusive event, *BMI* body mass index

**Table 11:** Concomitant medication use by adjudicated AOE and serious AOE status

	Total (*n* = 449)	No AOE (*n* = 371)	Any AOE (*n* = 78)	Serious AOE (*n* = 74)
Baseline concomitant medications				
Antihypertensives	86 (19)	63 (17)	23 (29)	22 (30)
Acetylsalicylic acid	39 (9)	23 (6)	16 (21)	15 (20)
Platelet aggregation inhibitors	38 (8)	22 (6)	16 (21)	15 (20)
Anti-diabetic agents	24 (5)	13 (4)	11 (14)	10 (14)
Lipid-modifying agents	22 (5)	16 (4)	6 (8)	6 (8)
Anticoagulants	15 (3)	13 (4)	2 (3)	2 (3)
Concomitant medication use at any time				
Antihypertensives	233 (52)	181 (49)	52 (67)	50 (68)
Acetylsalicylic acid	125 (28)	92 (25)	33 (42)	33 (45)
Platelet aggregation inhibitors	122 (27)	85 (23)	37 (47)	37 (50)
Anticoagulants	58 (13)	50 (13)	8 (10)	8 (11)
Lipid-modifying agents	51(11)	39 (11)	12 (15)	12 (16)
Anti-diabetic agents	45 (10)	26 (7)	19 (24)	18 (24)

Data are no. (%) of patients

*AOE* arterial occlusive event

The incidence of adjudicated AOEs by number of baseline risk factors (including arterial hypertension, hypercholesterolemia, obesity, diabetes mellitus, non-ischemic cardiac disease, and ischemic disease) is shown in Fig. 2C. The rate of adjudicated AOEs was 13% (24/189) among patients with 1–2 risk factors, and 29% (52/180) among patients with 3 or more risk factors. Of the 80 patients without any risk factors at baseline, only 2 (3%) had an AOE.

### Fatal AOEs

Separate adjudication of deaths revealed that 11 adjudicated AOEs were associated with death. These included 2 cases of cardiac arrest and 1 each of the following: bradycardic arrest, cardiac failure, intracranial hemorrhage, worsening of congestive heart failure, superior mesenteric artery occlusion, hemorrhagic cerebral infarction, congestive heart failure, ischemic stroke, and acute anterior myocardial infarction. Nine of the 11 patients with AOEs associated with death had a history of cardiovascular events and/or cardiovascular risk factors recorded at baseline (Table 12). The long-term survival of patients with adjudicated AOEs was similar to survival of patients without AOEs (Fig. 2D).

**Table 12:** Fatal AOEs and patient baseline characteristics

Fatal event	Fatal PT	Other AOE PTs reported	CML/ALL status	History of CV events	CV risk factors at baseline
Bradycardiac arrest	Cardiac arrest	Cardiac arrest Dry gangrene Peripheral ischemia	CML	Congestive heart failure Hypertension Impaired diastolic filling pattern Left atrium enlargement Mild tricuspid regurgitation Mitral valve calcification without significant mitral stenosis Intermittent ventricular tachycardia	Obesity Diabetes mellitus Arterial hypertension
Cardiac failure	Cardiac failure	Myocardial infarction Coronary artery disease Pulmonary embolism	CML	Pericarditis Ischemic heart failure	
Intracranial hemorrhage	Hemorrhage intracranial		CML	Aortic stenosis Calcified mitral annulus	
Worsening of congestive heart failure	Cardiac failure congestive	Myocardial infarction Deep vein thrombosis		QTc prolongation with nilotinib use Stent placement Congestive heart failure Myocardial infarction Coronary artery disease Mitral regurgitation Trace of tricuspid valve regurgitation	Hyperlipidemia Arterial hypertension
Superior mesenteric artery occlusion	Mesenteric arterial occlusion	Celiac artery occlusion	ALL	Paroxysmal atrial fibrillation Thrombophlebitis Bilateral leg deep vein thrombosis Cardiac catheterization	Hyperlipidemia Arterial hypertension
Cardiac arrest	Cardiac arrest	Peripheral vascular disorder	ALL	Greater saphenous vein thrombosis and cellulitis Aortic valve slightly thickened Left axis deviation Left bundle branch block Hypertension Mild aortic regurgitation Mild pulmonic valve regurgitation Mild to moderate tricuspid regurgitation	Arterial hypertension
Hemorrhagic cerebral infarction	Hemorrhagic cerebral infarction	Cerebral artery stenosis (2 events) Cerebral infarction (2 events)	CML		Diabetes mellitus Arterial hypertension
Cardiac arrest	Cardiac arrest		CML		
Cardiac arrest	Cardiac arrest		CML	Ischemic heart disease Angina pectoris	Coronary artery disease Type 2 diabetes mellitus Hypertension
Congestive heart failure	Cardiac failure congestive		CML		
Stroke	Cerebrovascular accident	Acute myocardial infarction (2 events)	CML	Ischemic stroke Ischemic heart disease Coronary artery disease Revascularization and coronary stent placement	Diabetes mellitus Arterial hypertension Hypercholesterolemia

*ALL* acute lymphocytic leukemia, *AOE* arterial occlusive event, *CML* chronic myeloid leukemia, *CV* cardiovascular, *PTs* preferred terms

## Discussion

In this study, adjudication of AOEs by an independent committee of experts allowed for a clinically meaningful description of AOEs associated with ponatinib, which can help to inform health care providers and patients of safety risks in an accurate and objective manner. The search that identified potential AOEs for adjudication was broader (based on 604 MedDRA terms related to vascular ischemia or thrombosis) than that initially used to calculate non-adjudicated AOE rates in the PACE trial (400 MedDRA terms) [3]. Based on 5-year follow-up of the PACE trial, the adjudicated AOE rate (17%) was lower than the non-adjudicated AOE rate (25%) [3]. Although the majority of adjudicated AOEs were serious, 81% of patients with AOEs continued on ponatinib (35% with dose modifications), the benefit of the drug was felt to outweigh the risk of the AOEs. Although vascular occlusive events were rarely reported during the initial development of second-generation BCR::ABL1 TKIs, a meta-analysis found that these events occurred in 5.9% of patients with CML treated with these agents, including bosutinib, dasatinib, nilotinib, and ponatinib [13]. In another review of prospective trials of patients treated with TKIs, including imatinib, nilotinib, dasatinib, and ponatinib, overall incidence of CV events was 45% (range, 41–63%) [14]. Accordingly, a high level of vigilance is indicated to recognize this potential complication of TKI therapy.

Notably, although concern existed around the potential for increasing AOE rates with long-term dosing, as seen with AEs related to other TKIs [15–18] the exposure-adjusted incidence of newly occurring adjudicated AOEs decreased over time on ponatinib, suggesting that the toxicity of ponatinib may not increase with longer treatment duration.

Patients with adjudicated AOEs were more likely to have multiple baseline cardiovascular risk factors (e.g., ischemic cardiac disease, arterial hypertension, hypercholesterolemia, and diabetes mellitus), and only 2 patients had an adjudicated AOE without any cardiovascular risk factors. These observations align with those of previous studies [6, 19]. It is important to identify and manage cardiovascular risk factors before and during therapy with ponatinib or other TKIs [20–22]. In PACE, 80% of CP-CML patients were resistant to dasatinib or nilotinib, and 24% had the *BCR::ABL1*^T315I^ resistance mutation [3]. Among CP-CML patients, estimated 5-year PFS and OS rates were 53% and 73%, respectively [3]. Data for overall survival in patients with and without adjudicated AOEs suggest that the risk of AOE-related death did not substantially impact survival, with disease-related death being the main driver of the OS curve. This underscores the need for providers to fully understand the therapeutic profile of ponatinib and consider its use when the potential benefits outweigh the risks for a given patient.

This study reinforces the importance of proper assessment of cardiovascular AEs to ensure accurate estimation of cardiovascular risk. The conventional processes of AE reporting and causality assessment may need to be re-assessed to avoid pitfalls associated with over- or under-reporting of AOEs, both of which may adversely affect patient care [23, 24]. Formal adjudication of events is a mainstay for development programs in other therapeutic areas such as diabetes mellitus [25, 26] and cardiology. A better understanding of the AOE risk associated with TKI therapy is a prime example of where formal adjudication is critical because accurate knowledge of risks is crucial before prescribing any TKI. The potential benefits of effective BCR::ABL1 TKI treatment, even with accompanying AEs, may outweigh the potential risks of progression-related mortality in patients with CP-CML and Ph+ ALL receiving second- or third-line therapy. This is particularly true for patients such as those with the *BCR::ABL1*^*T315I*^ mutation who may have limited treatment options [27]. Understanding the true incidence of the most significant events is a central element in properly assessing the benefit-risk ratio of an intervention. All later-generation TKIs are associated with risk of cardiovascular AEs [28], and the results of the formal adjudication process suggest the risk of these events with ponatinib may not be dissimilar to the event rates seen with some second-generation BCR::ABL1 TKIs [16–18].

A noteworthy finding in our analysis is that the exposure-adjusted incidence of newly occurring adjudicated AOEs decreased over time on ponatinib. These results are reassuring that the rate of new AOEs may not increase with longer duration of ponatinib treatment. Furthermore, patients with positively adjudicated AOEs were much more likely to have baseline cardiovascular risk factors (e.g., arterial hypertension, hypercholesterolemia, diabetes mellitus) or established cardiovascular disease; of those patients without any cardiovascular risk factors only 2 had a subsequent AOE. These results may provide clinical guidance with respect to the approach to use of ponatinib in patients at risk for an AOE. The ongoing phase 2 OPTIC trial (ClinicalTrials.gov Identifier: NCT02467270) is using a response-based dose reduction protocol approach to evaluate the optimal ponatinib dosing regimen for maximizing efficacy while mitigating toxicity. Results show that higher doses of ponatinib were associated with increased incidence of AOEs, with exposure-adjusted treatment-emergent AOE rates of 5.6%, 3.6%, and 2.1% for the 45-mg, 30-mg, and 15-mg cohorts, respectively [29]. However, the benefit differential was considerably larger with a starting dose of 45 mg, which was associated with a 26.3 percentage-point improvement in the response rate compared with a 15-mg starting dose (51.6% vs. 25.3%) [29]. Overall, the study indicated the best risk/benefit ratio when the 45-mg starting dose was reduced to 15 mg upon achievement of response (*BCR::ABL1*^*IS*^ transcript levels ≤ 1%) [29].

This retrospective study has strengths and limitations. The adjudication methodology provided a comprehensive and objective approach for characterizing AOE risk. A limitation is that only data from the clinical trial database were available. Prospective implementation of this strategy, as is being done in 2 ongoing trials, OPTIC and Ponatinib-3001 (NCT03589326), will overcome this challenge and add further value to the methodology and strength to the conclusions. In OPTIC, an independent cardiovascular endpoint adjudication committee is reviewing AOEs as they are reported using source documentation including cardiovascular workup (e.g., echocardiograms, electrocardiograms, biomarkers), hospitalization records, and any cardiovascular examinations performed.

## Conclusions

Independent reconsideration of AOEs by an expert adjudication committee showed lower rates of clinically relevant AOEs overall (17% vs. 25%) and serious AOEs (16% vs. 20%) than were originally reported in the PACE trial, suggesting an earlier possible overestimation that may not accurately reflect the AOE risk with ponatinib. The incidence of exposure-adjusted newly occurring AOEs decreased over time during ponatinib treatment. Improved understanding of the AOE profile with ponatinib and risk factors for AOEs can help guide decisions around TKI treatment. Results from the OPTIC study support a novel ponatinib treatment regimen of a 45-mg starting dose reduced to 15 mg upon achievement of response, maximizing response while minimizing toxicity [29].

## Data Availability

The data sets, including the redacted study protocol, redacted statistical analysis plan, and individual participants data supporting the results reported in this article, will be made available within three months from initial request, to researchers who provide a methodologically sound proposal. The data will be provided after its de-identification, in compliance with applicable privacy laws, data protection and requirements for consent and anonymization.

## Abbreviations

ACC: American College of Cardiology
AE: Adverse events
AHA: American Heart Association
ALL: Acute lymphoblastic leukemia
AOE: Arterial occlusive event
AP: Accelerated-phase
BP: Blast-phase
CML: Chronic myeloid leukemia
CP-CML: Chronic-phase chronic myeloid leukemia
MeDRA: Medical Dictionary for Regulatory Activities
PACE: Ponatinib Ph+ ALL and CML Evaluation
PFS: Progression-free survival
Ph+: Philadelphia chromosome positive
qd: Once daily
SCTI: Standardized Data Collection for Cardiovascular Trials Initiative
SMQ: Cardiac Failure Standard MedDRA Query
TKI: Tyrosine kinase inhibitor
