# Correction: Retrospective analysis of arterial occlusive events in the PACE trial by an independent adjudication committee

**DOI:** 10.1186/s13045-022-01239-x

**Published:** 2022-03-23

**Authors:** James L. Januzzi, Joseph M. Garasic, Scott E. Kasner, Vickie McDonald, Mark C. Petrie, Jonathan Seltzer, Michael Mauro, Kevin Croce, Ellin Berman, Michael Deininger, Andreas Hochhaus, Javier Pinilla-Ibarz, Franck Nicolini, Dong-Wook Kim, Daniel J. DeAngelo, Hagop Kantarjian, Jing Xu, Tracey Hall, Shouryadeep Srivastava, Daniel Naranjo, Jorge Cortes

**Affiliations:** 1 Massachusetts General Hospital, 55 Fruit Street, Boston, MA USA; 2 University of Pennsylvania, Philadelphia, PA USA; 3 Barts Health NHS Trust, London, England; 4 University of Glasgow, Glasgow, Scotland; 5 ACI Clinical, Bala Cynwyd, PA USA; 6 Memorial Sloan Kettering Cancer Center, New York, NY USA; 7 Brigham and Women’s Hospital, Harvard Medical School, Boston, MA USA; 8 Huntsman Cancer Institute, The University of Utah, Salt Lake City, UT USA; 9 Universitätsklinikum Jena, Jena, Germany; 10 H. Lee Moffitt Cancer Center & Research Institute, Tampa, FL USA; 11 Centre Hospitalier Lyon-Sud, Pierre-Bénite, Lyon, France; 12 Catholic Hematology Hospital, Seoul St. Mary’s Hospital, Leukemia Research Institute, The Catholic University of Korea, Seoul, South Korea; 13 Dana-Farber Cancer Institute, Boston, MA USA; 14 The University of Texas MD Anderson Cancer Center, Houston, TX USA; 15 Millennium Pharmaceuticals, Inc., A Wholly Owned Subsidiary of Takeda Pharmaceutical Company Limited, Cambridge, MA USA; 16 Georgia Cancer Center, Augusta, GA USA

## Correction to: Journal of Hematology & Oncology (2022) 15:1 https://doi.org/10.1186/s13045-021-01221-z

The original article contained errors in Figs. 1 and 2 which have since been corrected:

1. Figure 1 omitted sub-panel labelling.
2. Figure 2’s legend was inadvertently inverted.

